# Selective Stability Indicating Liquid Chromatographic Method Based on Quality by Design Framework and In Silico Toxicity Assessment for Infigratinib and Its Degradation Products

**DOI:** 10.3390/molecules28227476

**Published:** 2023-11-08

**Authors:** Awadh M. Ali, Mohammed M. Alanazi, Mohamed W. Attwa, Hany W. Darwish

**Affiliations:** Department of Pharmaceutical Chemistry, College of Pharmacy, King Saud University, P.O. Box 2457, Riyadh 11451, Saudi Arabia; aali1@ksu.edu.sa (A.M.A.); mmalanazi@ksu.edu.sa (M.M.A.); hdarwish@ksu.edu.sa (H.W.D.)

**Keywords:** Infigratinib, forced stress degradation, stability-indicating analytical methods, degradation products, quality-by-design, LC-MS/MS

## Abstract

Infigratinib, a protein kinase inhibitor employed in the therapeutic management of cholangiocarcinoma, was subjected to various stress conditions, including hydrolytic (acidic and alkaline), oxidative, photolytic, and thermal stress, in accordance with the rules established by the International Council for Harmonization. A cumulative count of five degradation products was observed. The application of the Quality by Design principle was utilized in the development of a rapid and specific separation method for Infigratinib and its degradation products. The methodology employed in this study was derived from an experimental design approach, which was utilized to examine the critical process parameters associated with chromatographic systems. The reversed-phase high-performance liquid chromatography technique, employing a C18 column and a mobile phase composed of a gradient mixture of 25 mM ammonium acetate buffer at pH 6.0 and acetonitrile, successfully facilitated the chromatographic separation. The methodology was expanded to include the utilization of UPLC-quadrupole tandem mass spectrometry in order to conduct a comprehensive analysis of the structural properties and characterize the degradation products. Overall, five degradation products were found in different stress conditions. The method was verified at certain working points, wherein a linearity range (5.0–200.0 µg/mL) was developed and other parameters such as accuracy, repeatability, selectivity, and system suitability were evaluated. Finally, the toxicity and mutagenicity of Infigratinib and its degradation products were predicted using in silico software, namely DEREK Nexus^®^ (version 6.2.1) and SARAH Nexus^®^ (version 3.2.1). Various toxicity endpoints, including chromosomal damage, were predicted. Additionally, two degradation products were also predicted to be mutagenic.

## 1. Introduction

**Figure 1:**
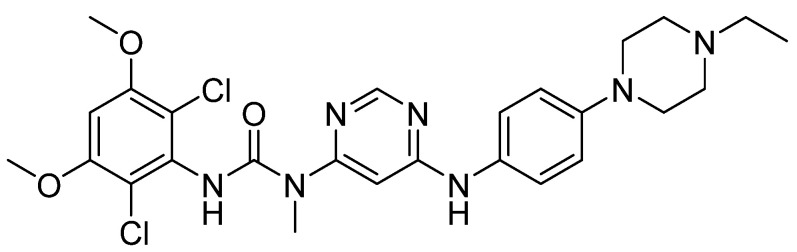
Chemical structure of Infigratinib.

Chemically, Infigratinib (INF) is 3-(2,6-Dichloro-3,5-dimethoxyphenyl)-1-(6-([4-(4-ethyl-1-piperazinyl)phenyl]amino)-4-pyrimidinyl)-1-methylurea (Figure 1). It is an orally bioavailable, human fibroblast growth factor receptors (FGFRs) reversible and specific inhibitor [1], which was recently approved for treatment of cholangiocarcinoma (bile duct cancer). Additionally, ongoing clinical trials are examining the function of INF in urothelial cancer, achondroplasia, and gastric cancer. Treatment with INF has potential negative side effects related to embryo-fetal toxicity, hyperphosphatemia leading to soft tissue mineralization, and retinal pigment epithelial detachment (RPED) [2].

Different studies are available related to INF analysis, including pharmacodynamics [2], pharmacokinetics [2,3,4], and metabolic profiling [5,6,7]. Some are with toxicity investigation, which is an integral part of drug development and quality assurance for the metabolites produced. Stability of pharmaceutical substances and products is crucial to maintain their identity, purity, and potency within predetermined limits for a specified amount of time. Stability testing of a drug substance may include tracking the development of degradation products (DPs), in addition to the loss of the active pharmaceutical ingredient (API) [8]. A stability study is mainly implemented to test with time the quality of a drug substance or drug product under the influence of a diversity of climatic factors such as humidity, pH, light exposure, and temperature, and to determine retesting periods, shelf lives, and recommended storage conditions for drug substances and products [9].

A one-factor-at-a-time (OFAT) methodology has been used to create stability-indicating analysis methods (SIAMs) repeatedly. The validation step of the OFAT technique often yields many unexpected outcomes, among them the fading out and/or emergence of a few degradation products, particularly when the stress sample involves many DPs. This method may not produce a reliable SIAM method and is exceedingly costly, tedious, and time-consuming, and one might have to return to the development stage at the end. Therefore, to expedite the method creation process, it is vital to establish a systematic and logical approach with a limited number of trials. Quality by design (QbD) is a systematic approach based on the design of experiments (DoEs) and begins with predefined objectives. This approach will be adopted in our study to obtain adequate knowledge of the efficiency and safety of INF and its DPs. It is therefore needed to assess drug quality at all stages of development by understanding and analyzing complex processes and converting data into knowledge. Many regulatory parties, including the International Council for Harmonization (ICH), have representative guidelines that are based on QbD, such as Q8 “pharmaceutical development” and Q9 “quality risk management.” [10,11]. This means that QbD can significantly reduce out-of-trends, out-of-specifications results, and product failure, which consequently leads to high-quality products. This is because it produces a robust method with fewer resources for drug evaluation and analysis [12]. The QbD technique enables us to scientifically observe the critical method variables by bringing a scientific understanding of the method variables to the method response. QbD overcomes the traditional OFAT limitations by designing experiments relying on multivariate analysis. In order to detect chromatographic selectivity and promote an improved method control strategy, regulatory authorities have been urging and encouraging analytical scientists to investigate the QbD methodology [11,13]. According to this viewpoint, design space (DS) plays a significant role in the process of creating QbD-based analytical methods. A space of chromatographic conditions that guarantees the high quality of each analyte’s separation may be thought of as DS for chromatographic procedures. A procedure is hence reliable within the DS bounds.

By simultaneously examining all critical chromatographic process parameters, such as gradient duration, column oven temperature, flow rate, and mobile phase pH using DoE, DS may be produced [11,14]. To the authors’ best knowledge, no analytical method has been developed and published yet for the stability study of INF and its DPs and their potential toxicity evaluation. As a result, this study aimed to develop and validate a stability-indicating method that can distinguish INF from its associated DPs in forced stress conditions [15,16,17,18,19]. High-performance liquid chromatography (HPLC) and LC-mass (LC-MS) techniques were utilized to characterize and identify the INF and its DPs. Finally, in silico studies were employed to test the potential toxicity and mutagenicity of the INF and its DPs utilizing computational methods, specifically the DEREK Nexus^®^ (version 6.2.1) and SARAH Nexus software^®^ (version 3.2.1). This study utilized a comprehensive array of specialized software (DEREK Nexus^®^) for the assessment of many toxicity endpoints, including carcinogenicity, genotoxicity, mutagenicity, teratogenicity, irritating effects on the skin, allergic reactions, and impacts on fertility. It has the potential to be utilized as a component within an ICH M7 workflow [20]. In another in silico statistical model, called SARAH Nexus^®^, the prediction of bacterial in vitro reverse mutagenicity, namely in the Ames test [21], depends on taking into account statistical factors such the structural similarity and Ames result of data points in the predictive model. By comparing structural segments shared between the input molecule and molecules in the dataset, SARAH produces predictions. After this analysis, a numerical calculation is carried out to establish how confidently a particular fragment is connected to bacterial mutagenicity [22].

## 2. Results and Discussion

The creation of a QbD-based analytical method may be broken down into four steps: (a) the method analytical target profile (ATP); (b) critical quality attributes (CQAs); (c) risk assessment parameters using DOE (screening, prioritization, and optimization); and (d) defining design space (DS).

### 2.1. ATP of the Method

ATP is comparable to the method’s objectives, which were to separate the INF and DPs with a resolution greater than 1.5 (baseline separation). For the identification of low levels of DPs during routine drug analysis, the method’s sensitivity and selectivity are also very important. Finally, for choosing a common detection wavelength for the drug and DPs, the PDA detector was used to assess the UV spectra of the peaks from 200.0 to 400.0 nm.

### 2.2. CQAs of the Method

CQAs are requirements to maintain drug quality within a proven acceptable range or limit for identification, separation, accuracy, precision, robustness, and ruggedness. Resolution (R_s_), capacity factor, column efficiency, retention time, peak tailing of the analytes, and precision are selected CQAs of the procedure for chromatographic techniques. The number of peaks with a resolution greater than 1.5 and the total number of peaks were chosen as CQAs in the current study and were directly modeled using a multivariate model with Design Expert^®^ modeling software (version 12).

### 2.3. Risk Assessment “Screening, Prioritization, and Optimization”

Risk analysis is an essential component of the QbD approach. It is primarily concerned with identifying and rating method factors that affect the method’s performance and ATP conformity. Risk assessments are often carried out throughout the method development process, paying attention to potential variations in lab procedures and reagent supplies. The risk management strategy directs our efforts and resources to the places where they are most needed. In HPLC method development, risk assessment will involve qualitative variables such as type of organic phase, buffer, column, detector, and elution mode (isocratic or gradient) and quantitative variables such as % of aqueous, % of modifier, pH, flow rate, and column temperature.

DoE will provide an efficient and competent way of evaluating the effects of all components together with their interactions and forecasting the link between these factors and CQAs to optimize a chromatographic procedure. To ascertain the impact of one component when there are other factors present, it is important to simultaneously examine all critical parameters instead of separately. However, the full factorial design of all parameters results in enormous experiments. Therefore, the risk evaluations were conducted in two stages. The first stage involved screening the primary factors that affect resolution (such as gradient time and column type) using the traditional approach of OFAT. The second stage involved an optimization study in the presence of other influencing parameters (such as flow rate, pH, organic modifier, and column temperature) using DoE.

### 2.4. DS “Region of Operable Method”

The region of operable method is a multidimensional region where quality is assured due to the integration and interaction of input variables. The design space will bring about a product that will fulfill the stated quality standards. It enables the analytical procedure to change toward being more effective or less risky, depending on the design of the analytical equipment. Therefore, for an HPLC method development of a drug substance that undergoes degradation in various conditions, the design space to manage the separation process (e.g., No. of peaks and No. of peaks with resolution greater than 1.5) might be represented for each unit operation or as a total over all unit operations. DoE from Design Expert^®^ modeling software (version 12) is used to create the design space for screening experiments, regression modeling, response surface modeling, comparison experiments, and mixture studies.

### 2.5. Screening and Optimization

#### 2.5.1. Screening Experiments

**Figure 2:**
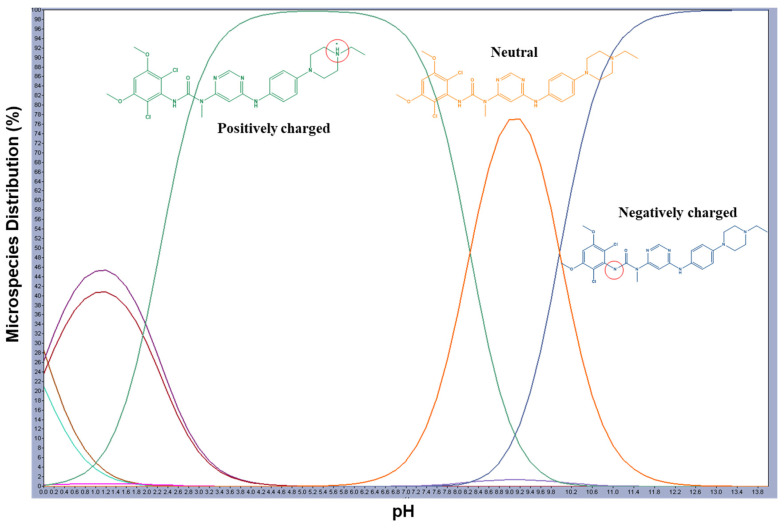
Infigratinib’s predicted pH curves overlaid with the three most common microspecies, created using MarvinSketch. The ionized portions of the molecule are shown with a red circle. Green for positively charged, orange for neutral, and blue for negatively charged ions.

Preliminary tests were carried out based on the knowledge gained through the literature about the molecule itself. Part of the method screening consisted of multiple OFAT experiments. Reversed-phase LC was chosen based on the molecule structure and characteristics of the expected DPs. To avoid tailing, which could happen if the molecule shifted from one ionized form to another at the chosen mobile phase pH since ionization affects the retention of the molecule on the stationary phase, it is preferable to be in a single ionized form at the chosen pH for LC. According to the MarvinSketch (ChemAxon, Budapest, Hungary) anticipated pH curves (Figure 2) [23], INF demonstrates a wide variety of species over the pH range. Three alternatives were available: a pH around 5.2, a pH around 9.2, and a pH at 13.0, where the INF will be >90% in a single ionized form. The choice was to examine an aqueous portion of the mobile phase with an acidic pH because the basic pH of 9.2 or, more aggressively, pH 13.0 is often not applicable with most reversed-phase chromatographic columns. Additionally, to maintain MS compatibility, ammonium acetate was chosen as the buffer as it is compatible with MS procedures used later to characterize DPs. ACN was first chosen as the organic phase to be tested. Gradient elution is the best option to separate INF and its DPs from one another due to the anticipated distinct polarity of each.

To better understand at what rate INF elutes from the C_18_ column and to increase selectivity between INF and its DPs, a low to high organic phase percent was tested utilizing forced degradation samples that had already decayed and the substance solution for INF. First, compared to INF, a significant degradation product is more polar and requires less ACN to elute in a timely manner; at >20% ACN, it will elute in the solvent front. Nonetheless, more organic phase is required for INF to elute from the column. Second, we are looking for minor peaks of degradation products that will be obscured by a rapid change in the slope; hence, the change in the mobile phase slope should be slow. This requires additional gradient time. The optimal gradient time was selected as mentioned under liquid chromatographic conditions.

Additionally, raising the pH to 6.0 leads to greater component separation, but the question was how much higher the pH could be raised before it started to impact the solubility of the stationary phase. As a result, optimization with QbD was used to determine the critical operating parameters for chromatographic separation.

#### 2.5.2. Optimization Experiments

**Figure 3:**
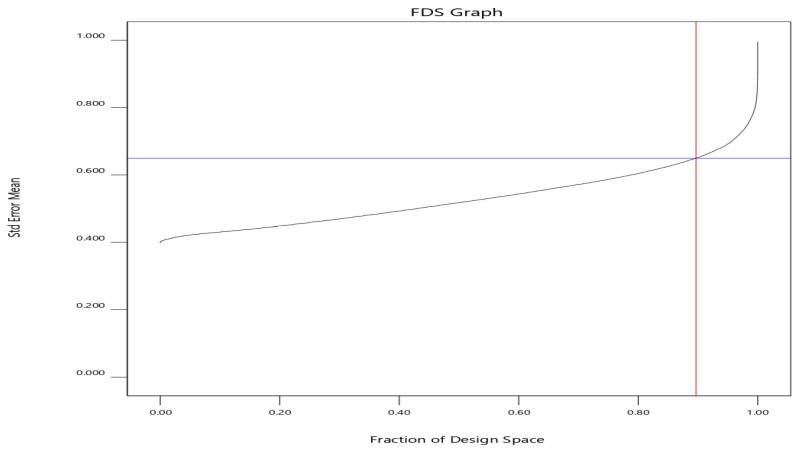
Fraction of design space of the developed model. Black line indicates the fraction with a value more than 90% (red vertical line) as a function of standard error of the mean with a value less than 0.644 (blue horizontal line).

To determine the ideal chromatographic condition and examine the interactions of these parameters collectively, the chromatographic conditions were analyzed using an optimal randomized response surface design with three numeric factors (buffer pH, flow rate, and column temperature) and one categorical factor (organic modifiers ACN and MeOH). A total of 31 experiments were conducted using these parameters at various levels (pH 5.5–6.5; flow rate 0.8–1.2; column temperature 25–50 °C) and two levels for categorical. Based on the model’s evaluation prior to experimental activity, the variance inflation factors (VIFs), which calculate how much the variance of a model coefficient rises as a result of the design’s lack of orthogonality, a coefficient’s VIF is one if it is orthogonal to the other model terms. It is less than twice the average values for the leverages of design points, which when increased will negatively affect the model fit, and a correlation of model coefficients of zero, which denotes the orthogonality of the model. Finally, it is worth noting that the fraction of design space (FDS) provides a means of visually assessing the accuracy capability of a given design. Lower average error scores and more constant error ratings across the factor space are indicators of a stronger design. The graphs demonstrate a decrease in the curvature of the curves, indicating a more flattened shape (Figure 3). This characteristic is employed to ascertain the extent of the design space, with a prediction variance of less than 0.644 and a 90% observed percentage in this particular design. All of these parameters collectively indicate a well-selected design.

MeOH and ACN were chosen as organic modifiers due to the fact that their effects on separation, which were well documented through the literature, prompted their selection. To achieve good technique selectivity and enhanced analyte peak shapes, the mobile phase’s pH must be carefully managed. Thus, various continuous pH 5.5–6.5 levels were employed. The selected buffers were chosen to ensure compatibility with MS. Ammonium acetate (25 mM) was utilized as a buffering agent. The alteration of flow rate can also exert an influence on the process of separation. Consequently, within the practical constraints of the HPLC instrument, adjustments to the flow rate were made within the range of 0.8–1.2 mL/min. Numerous studies have demonstrated the influence of temperature on HPLC separation. Therefore, a temperature range of 25–50 °C was selected for implementation in this experimental design.

The buffer pH, flow rate, column temperature, and organic modifier combination that produces the greatest number of integrated peaks and peaks with a resolution (Rs) ≥1.5 was determined by evaluating the mixture of the INF and DPs from all stress situations under various settings and using the overall selectivity. The planned optimization approach necessitates the execution of numerous experiments, including duplicates, blanks, and column re-equilibration between runs. An optimal separation technique should possess sufficient chromatographic efficacy and demonstrate the capability to effectively separate INF from its DPs, as well as separate the DPs from each other.

Based on UV spectra and the area of each peak, the INF and DPs peaks were tracked. The analysis covered 31 chromatograms from optimization experiments (Figure S1). The chromatograms were accurately processed using LCsolution software (version 1.25) and subsequently transferred to the modeling software, Design Expert^®^ (version 12). The data that were imported were subjected to analysis using modeling software. This analysis led to the selection of the quadratic model as the most suitable model for accurately representing the number of peaks with a United States Pharmacopeia (USP) resolution of 1.5 or higher (which was six peaks under optimized conditions in our work). The chosen model was deemed significant based on a *p*-value of less than 0.0001. Additionally, the quadratic model exhibited an adjusted R^2^ value of 0.9828 and a predicted R^2^ value of 0.9472. Based on the ANOVA analysis, it can be concluded that the model is statistically significant, as indicated by the F-value of 133.10. This suggests that the likelihood of observing an F-value of this magnitude due to random variation is extremely low, at a significance level of 0.01%. Table 1 presents the significant model terms, which include the organic modifier, temperature, as well as the interactions between pH and temperature, pH and organic modifier, pH and flow rate, pH and temperature, pH and organic modifier, flow rate and temperature, and temperature and organic modifier. Additionally, certain individual squared factors are also found to be significant. The equation, expressed in terms of coded factors, is presented as follows:

The equation can be written as follows:

$$Y=4.07+0.0810A+0.0755B-0.2161C+1.10D+0.2632AB-0.1201AC-0.1055AD-0.1371BC-0.0712BD\;-0.2729CD-0.4152A^{2}-0.4599B^{2}+0.7549C^{2}$$

**Figure 4:**
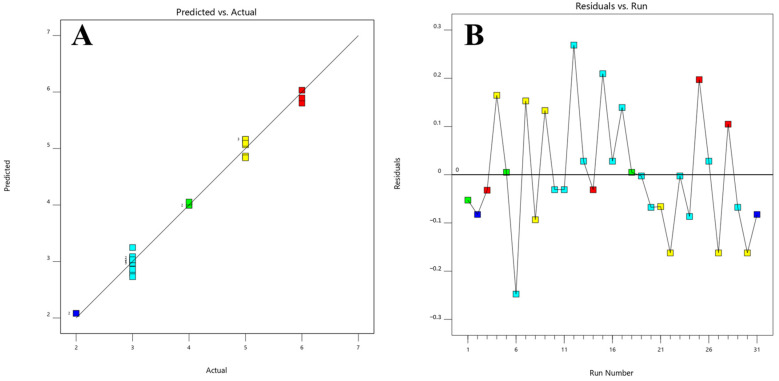
Model diagnostics plots of the number of peaks with a resolution ≥1.5 response. (**A**) The predicted response values vs. the actual response values; (**B**) the residuals in relation to the experimental run order. Different colors indicate different runs.

**Figure 5:**
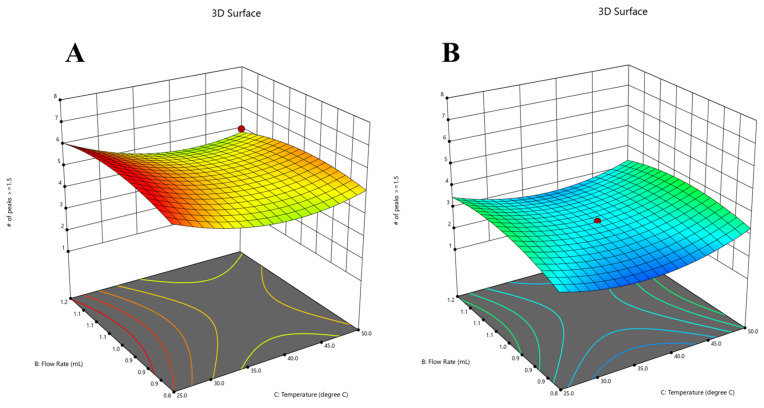
The 3D surface model at a pH of 6.0 in the two different organic phases, ACN (**A**) and MeOH (**B**), specifically for the number of peaks with a resolution ≥1.5 response.

Figure 4 displays the graphical representations of the model’s predicted and residual values plotted against the actual and run variables, respectively. The utilization of ACN as the organic modifier under lower temperature conditions resulted in the highest number of peaks compared to MeOH, while maintaining a stable pH of 6.00. This observation is depicted in Figure 5, which presents a 3D surface representation. The color code employed in the image visually represents the numerical values of the number of peaks achieved with a resolution of 1.5 USP. The warm hues, spanning from the color red to orange, are indicative of the maximum peak values, which align with a resolution of six peaks. On the other hand, the cool blue hues are indicative of the minimum values.

**Figure 6:**
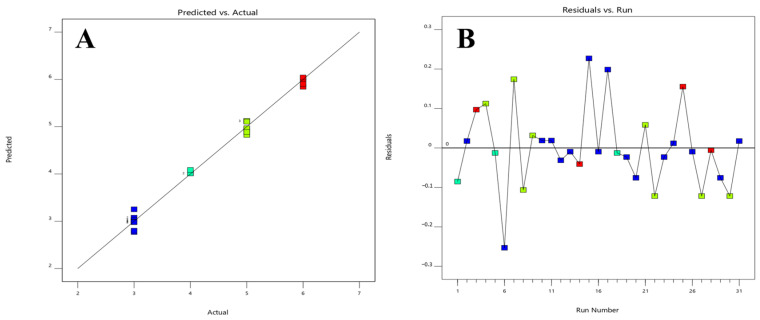
Model diagnostics plots of the total number of peaks response. (**A**) The predicted response values vs. the actual response values; (**B**) the residuals in relation to the experimental run order. Different colors indicate different runs.

**Figure 7:**
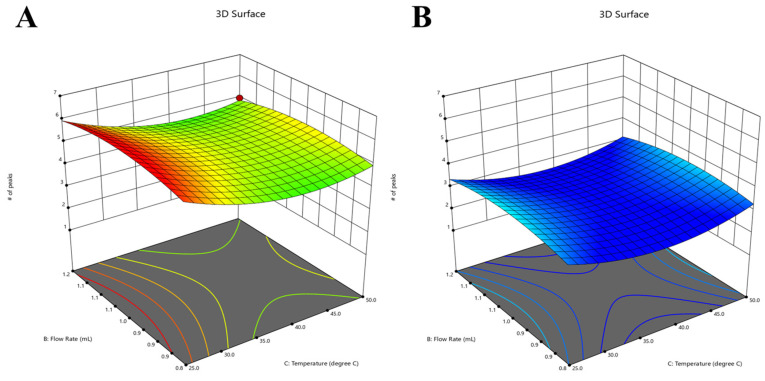
The 3D surface model at a pH of 6.0 in the two different organic phases, namely ACN (**A**) and MeOH (**B**), specifically for the total number of peaks response.

The model that best fits the data for the number of peaks response is also quadratic, with a significant *p*-value and an adjusted R^2^ value of 0.9860. Additionally, the predicted R^2^ value for this model is 0.9551. The obtained model F-value of 164.03 indicates that the model is statistically significant. The probability of observing an F-value of this magnitude solely owing to random variation is extremely low, at 0.01%. The relevant model variables in Table 2 include the organic modifier, temperature, pH, and flow rate interaction, temperature and organic modifier interaction, as well as some individual squared factors, which are significant model terms. Figure 6 displays the graphs depicting the comparison between the predicted values and the actual values, as well as the residuals plotted against the run. The number of peaks achieved at lower temperatures is greater when using ACN as the organic modifier compared to MeOH while maintaining a fixed pH of 6.00. This observation is depicted in Figure 7, where a 3D surface plot is utilized, employing color coding to graphically convey the numerical values corresponding to the number of peaks. The warm hues, including a spectrum from red to orange, serve as indicators of the most elevated peak values, aligning with a resolution of six peaks. In contrast, the frigid blue hues are indicative of the minimum values. The equation expressed in terms of coded factors is presented below:

(1) $$Y=4.07-0.0180A-0.0106B-0.2763C+1.06D+0.4007AB-0.0137AC-0.0106AD-0.0401BC+0.0183BD\;-0.2118CD-0.2890A^{2}-0.3833B^{2}+0.6696C^{2}$$

**Figure 8:**
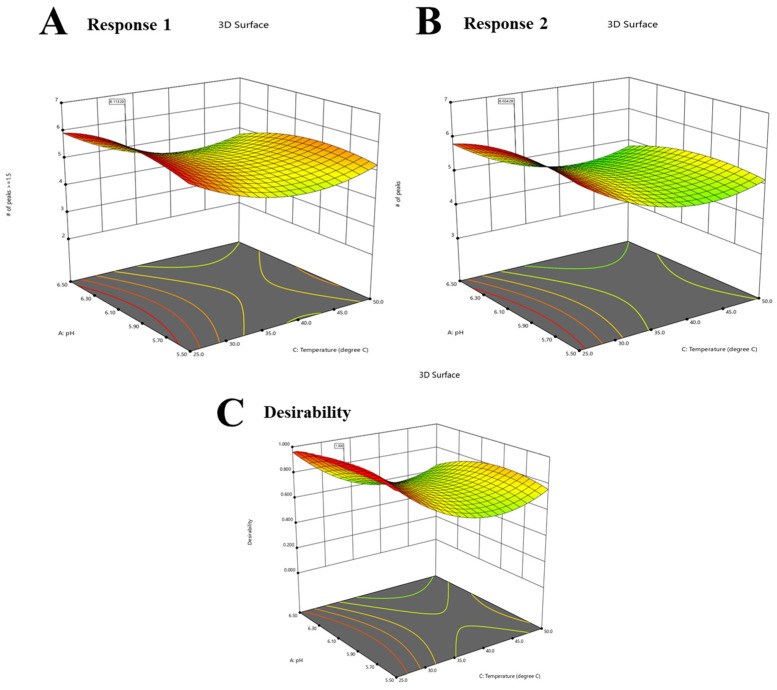
Numerical optimization to response 1 (the number of peaks with a resolution ≥1.5) (**A**). Numerical optimization to response 2 (the total number of peaks) (**B**). The optimal desirability of both responses (1 and 2) (**C**). The flow rate was 1 mL/min and pH was 6.0.

The numerical optimization of the evaluated replies aims to achieve the maximum values for each individual response while also striving for a desirability close to 1, as illustrated in Figure 8.

#### 2.5.3. Design Space

**Figure 9:**
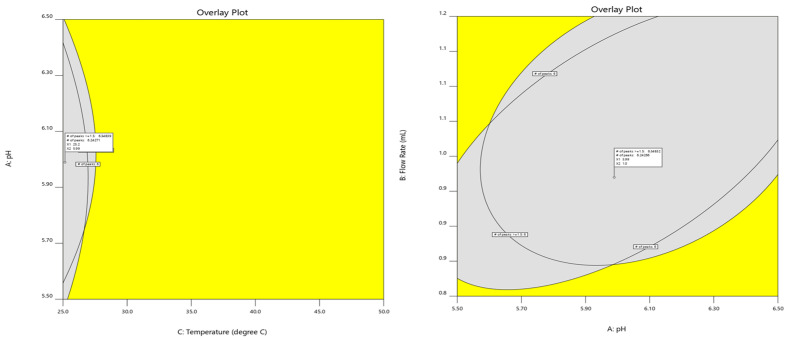
Overlay plot of graphical optimization where the specified requirements are met. For the best outcomes, the number of peaks with a resolution ≥1.5, and the total number of peaks in general, both responses were maximized.

The overlay graph represents a singular plot that emphasizes the optimal region where response requirements can be simultaneously satisfied for both responses. Figure 9 is utilized to demonstrate the boundaries of the operable zone in collective responses. The utilization of ACN at a temperature of 25 °C, a flow rate of 1.0 mL/min, and a pH value of 6.00 is situated inside the designated region of the design space.

### 2.6. Degradation Behavior of the INF

Figure 4 displays the ultimate chromatograms, representing a composite of all stress degradation samples. To confirm the presence of DPs, each sample experiencing stress degradation was injected individually. The drug exhibited five distinct degradation products (DPs), namely D1, D2, D3, D4, and D5, when subjected to various stress conditions.

**Figure 10:**
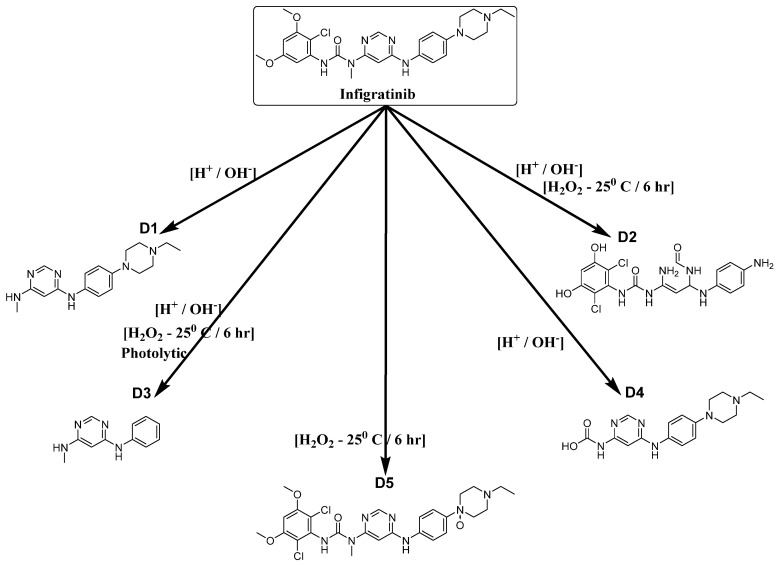
The proposed degradation pathway of Infigratinib under various forced stress conditions.

The INF was subjected to amide hydrolysis under the various stress conditions previously described. This process resulted in the formation of a predominant DP (referred to as D1) that was detected and eluted with a retention time of 6.3 min. The percentage observed under all stress circumstances ranged from 2.24% to 11.1% (Figure 10). A secondary DP (D2) was observed in lower concentration levels ranging from 0.09% to 0.15% across all stress conditions, arising from O-demethylation, hydrolysis of 2-heterosubstituted pyridine followed by ring opening, and amine hydrolysis of tertiary aliphatic amine. The presence of DP D3 was solely detected in minimal quantities (0.005–0.014%) exclusively under instances of oxidative and photolytic stress. It is a consequence of the hydrolysis of amides and amines. The hydrolysis of amide also results in the generation of an additional DP (D4), which has been detected under conditions of acidity, alkalinity, and oxidative stress. Finally, the production of *N*-oxide (D5) occurred exclusively under oxidative conditions, as a consequence of the *N*-oxidation of an aliphatic tertiary amine. The proportions, expressed as percentages, of the total area attributed to each DP are displayed in Table 3.

### 2.7. UPLC-TQD-MS/MS for the Characterization of DPs

**Figure 11:**
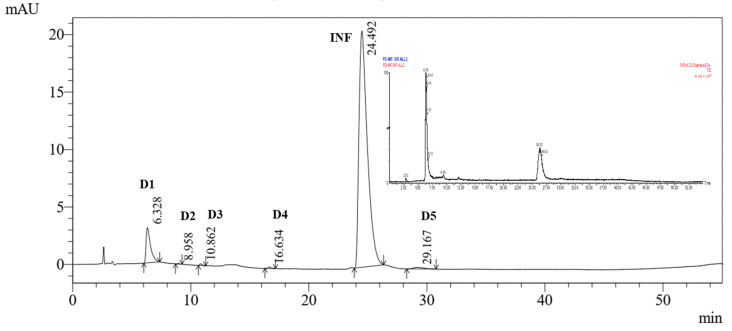
UV chromatogram at flow rate of 1.0 mL/min and pH of 6.0 of the mixture showing the selective ion monitoring (SIR) mass spectrum of the Infigratinib and its degradation products under different stress conditions.

The degradation of INF was seen to occur in many DPs when subjected to various stress conditions, including hydrolytic, thermal, and photolytic stress. Characterizing and identifying all the DPs that are not present in their pure form is a challenging undertaking. Consequently, a deliberate attempt was undertaken to define the DPs through the utilization of online UPLC-ESI MS/MS and utilizing the fragmentation pattern (Table 4 and Figure 11).

#### MS/MS Fragmentation Pattern of Protonated INF

**Figure 12:**
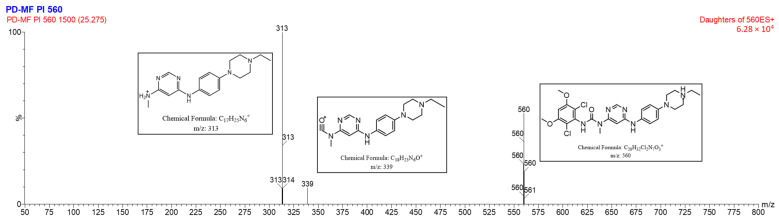
ESI-MS fragmentation spectrum of ([M + H]^+^) ion of INF (*m*/*z* 560).

The ESI-TQD spectrum of INF exhibited a peak in positive mode corresponding to a protonated molecular ion at *m*/*z* of 560. The chemical formula of this ion was determined to be C_26_H_32_Cl_2_N_7_O_3_^+^. ESI-MS/MS analysis of the protonated molecular ion ([M + H]^+^) of INF revealed the presence of product ions at *m*/*z* 339 and *m*/*z* 313, as seen in Figure 12. The predicted fragmentation pathway of the drug was determined by analyzing the MS/MS data in conjunction with the most likely formulas of the product ions, which were generated from the *m*/*z* readings. The fragmentation of the amide bond inside the original molecule results in the generation of a product ion with an *m*/*z* of 339. The formation of the base peak at *m*/*z* 313 can be attributed to the breakage of the second amide bond. The structural representation of the fragment ions of DPs are depicted in Figures S2–S6.

### 2.8. Validation of the Developed Method

The SIAM that was developed underwent validation in accordance with ICH guideline Q2 (R1). The method’s selectivity was assessed by analyzing the peak purity of INF with a PDA detector. The study revealed that the purity of the analyte was deemed suitable, and the procedure employed demonstrated selectivity (Figure S7). The linearity of the data was confirmed across the concentration range of 5.0–200.0 µg/mL. The data from the calibration curve underwent statistical analysis utilizing a linear regression model. The resulting linear regression equation and correlation coefficient were determined to be y = 20,887x – 121,902 and 0.9998, respectively.

The precision of measurements within a single day and across three days was assessed at three distinct concentrations: 15.0, 80.0, and 130.0 µg/mL. Each concentration was tested in triplicate on both the same day and on successive days. The intraday and interday precision experiments yielded %RSD values of less than 2% (Table 5), indicating that the proposed approach exhibits a high level of precision. The accuracy of the INF assay was assessed by analyzing a standard sample at five distinct concentration levels: 15.0, 30.0, 80.0, 130.0, and 180.0 µg/mL. Each concentration level was replicated three times to ensure reliability and minimize experimental error. The range of %RSD values was determined to be 0.26–1.06%. System suitability parameters’ results are represented in Table 6.

The method’s robustness was assessed to ascertain the study’s dependability in the face of intentional alterations to the parameters of the methodology. By employing the QbD methodology and utilizing the Design-Expert software (version 12), a resilient zone was successfully found without the need for additional experimentation. The robustness of the projected robust region was assessed by picking three confirmation locations inside the design space. It was observed that the response of all peaks remained unaffected despite variations in the technique parameters. This finding suggests that the approach demonstrated robustness within the specified design space, as shown in Table 7.

### 2.9. In Silico Toxicity and Mutagenicity Prediction of INF and Its DPs

The toxicity and mutagenicity of INF and its DPs were evaluated utilizing the DEREK (version 6.2.1) and SARAH software tools (version 3.2.1). Table 8 presents the results of the toxicity and mutagenicity studies performed on INF and its DPs. In order to create predictions, a range of criteria, including humans, monkeys, pigs, dogs, rabbits, guinea hamsters, mice, primates, rats, bacteria, and Salmonella typhimurium, were used. Several endpoints were computed, encompassing carcinogenicity, skin sensitization, teratogenicity, hepatotoxicity, chromosomal damage, neurotoxicity, phospholipidosis, nephrotoxicity, phototoxicity, and more endpoints. The investigation conducted by DEREK sought to compare the toxicity predictions of each DI with the INF. The following conclusions are here: The compounds INF, D1, and D4 were anticipated to induce chromosomal damage, phospholipidosis, skin irritation, and skin sensitization. These effects can be attributed to the presence of para-phenylenediamine, aryl piperazine, alkyl amine, and both diamine and amino-substituted aniline moieties in the compounds, respectively. D2 was predicted to induce chromosomal damage, hepatotoxicity, thyroid toxicity, and skin sensitization as a result of the inclusion of para-phenylenediamine, formamide derivatives, resorcinol, and both amino-substituted aniline and resorcinol, respectively. No alarms for toxicity were predicted in D3. The presence of alkyl amine in D5 was predicted to result in skin irritation.

The outcomes of the INF and its DPs (D2, D5) have demonstrated negative findings for the in vitro mutagenicity endpoints as predicted by the SARAH tool. The results of this study indicate that the likelihood of these substances inducing mutations is unlikely. Nonetheless, the data from D1, D3, and D4 demonstrated positive outcomes in relation to potential mutations. In a broader context, these scenarios possess inherent value in the inspection of the deteriorating conditions of the INF.

## 3. Experimental

### 3.1. Material and Reagents

INF standard (DD-061218; ≥98%) was supplied by LEAPChem (Hualong, Hanzhou, China). Dimethyl sulfoxide (DMSO, 99.9% (CH_3_)_2_SO); Fisher, Oxford, UK), ammonium acetate (CH_3_COONH_4_, UK), acetic acid (99.7% CH_3_COOH; Winlab, Market Harborough, UK), hydrochloric acid (36% HCl *w*/*w*; Fluka, London, UK), sodium hydroxide pellets (NaOH; Merk, Darmstadt, Germany), and hydrogen peroxide (30% H_2_O_2_ *w*/*w*; Avonchem, London, UK), all of analytical reagent grade, were purchased from the local market. Acetonitrile (ACN, C_2_H_3_N) and methanol (MeOH) of HPLC grade were purchased from Sigma-Aldrich Company (West Chester, PA, USA). Ultrapure water was purified in situ with a Milli-Q Plus filtration system from Millipore (Millipore, Bedford, MA, USA).

### 3.2. Instrumentation

For the separation, identification, and quantitative determination of INF and its associated DPs, an HPLC equipped with a diode array detector (PDA) was used (Shimadzu, LC-20AD, Japan). The C_18_ column (Zorbax Eclipse Plus C_18_, 250 mm, 4.6 mm, 5 µm; Agilent, Santa Clara, CA, USA) was the stationary phase that was installed. LCsolution software (version 1.25) was used to monitor and process the output signal. An ultrasonic bath (Elma S180H; Singen, Baden-Württemberg, Germany) and pH-meter (pH 211; Hanna, Nusfalau, Romania) were used to prepare the samples and measure the mobile phase pH, respectively. Shaker apparatus (Maxi-shake; Heto, Allerød, Denmark) was employed to facilitate the process of heat-mediated hydrolysis. An oven (Genlab, Halton, UK) was employed to conduct forced degradation under controlled temperature conditions. An analytical balance (model B154-S; Mettler Toledo, Greifensee, Switzerland) was used to weigh samples and standards. In accordance with option 2 of the ICH guideline Q1B [24], photolytic studies were conducted in a photostability chamber (APT.line^®^ KBF-ICH-720; Binder, Tuttlingen, Germany) that was set at 40 ± 5 °C/30%RH ± 3%RH and equipped with ICH-compliant illumination in the door made up of a combination of UV and white fluorescent lamps. On a Waters UPLC-MS/MS instrument harboring Acquity UPLC (H10UPH) and Acquity TQD MS (QBB1203) combined with electrospray ionization (ESI), LC-MS/MS investigations were conducted. The C_18_ column was used as the stationary phase. The fragmentor voltage was set to 16 V, the capillary voltage to 30 V, the capillary temperature to 250 °C, and the source nitrogen gas flow was set to 650.0 L/h after tuning of MS parameters with IntelliStart^®^ software (version 4.1, SCN 805) for INF. These were the ideal mass detector operating settings for the LC-MS/MS study. As the collision gas, extremely pure nitrogen gas from a nitrogen generator (Peak Scientific, Inchinnan, UK) was employed. For the fragmentation of analyte ions into relative fragments, argon (99.999% in cylinders) was used as a collision gas in the TQD mass analyzer. The required vacuum for the TQD mass analyzer was generated using a vacuum pump (Sogevac^®^; Murrysville, PA, USA). All the spectra were captured under the same experimental circumstances with MassLynx 4.1 software (Version 4.1). Additionally, two HPLC columns were tested: Phenomenex C_18_ (Torrance, CA, USA), Grace phenyl, and Cyano, which had dimensions of 250 × 4.6 mm, 5 µm (Bannockburn, IL, USA).

### 3.3. Software

The optimization of pH, temperature, and organic modifier to separate a mixture of INF from its DPs was carried out using Design Expert^®^ modeling software (version 12; StatEase Inc., Minneapolis, MN, USA).

Lhasa Limited has developed two software tools, namely DEREK Nexus^®^ and SARAH Nexus^®^, which are specifically created for the evaluation of chemical toxicity and mutagenicity. SARAH Nexus^®^ is a tool that uses statistical methods and machine learning techniques, whereas DEREK Nexus^®^ is a tool that applies a knowledge-based approach to produce predictions. DEREK Nexus^®^ may be utilized to estimate the potential hazards of a chemical by assessing numerous endpoints, including mutagenicity, teratogenicity, carcinogenicity, skin irritation, hepatotoxicity, and phototoxicity across different species. The accuracy of the results obtained using SARAH Nexus^®^ is notably high since it relies on a comprehensive dataset of compounds that have undergone thorough scrutiny in terms of their mutagenic properties. The chemical structure of INF, together with each DI, was loaded separately, and a setup command was executed to initiate the predictions of DEREK and SARAH. Explanations of the predictions are provided in some detail in Section 2.9.

### 3.4. Sample Preparation and Stress Degradation Study

The process of finding a suitable solvent for sample preparation has proven to be a substantial problem. In ACN and MeOH, which are frequently employed in the preparation of reversed-phase LC samples, INF is poorly soluble in these solvents. It is also almost completely insoluble in water (<0.1 mg/mL) but soluble in DMSO [25]. Furthermore, using DMSO as the only solvent gives an intense peak in HPLC, which covers the drug’s main peak and has a high UV cutoff point. Because of this, the first attempt to dissolve INF in 40:60% DMSO: ACN succeeded. ACN was used to optimize the water content in the solvent, reducing the solvent elution impact in liquid chromatography, which might lead to an undesirable peak shape. In order to enhance solubility, a sonication process lasting 5 min was employed in the solvent.

A standard stock solution (2 mg/mL) of INF was prepared by dissolving 50 mg of the INF standard in a 25 mL volumetric flask and dissolving in a diluent (40:60% *v*/*v* mixture of DMSO and ACN, respectively), sonicating for 5 min, and then completing to the mark with the same diluent. This solution was stored in a freezer (−20 °C) until needed.

### 3.5. INF’s Forced Degradation Study

According to the ICH (Q1A (R2), Stability Testing of New Drug Substances and Products) and Q1B (Photostability Testing of New Drug Substances and Products) recommendation [9,24], INF was subjected to hydrolytic (acidic and basic), oxidative, thermal, and photolytic conditions during the stress degradation. A final concentration of 1.0 mg/mL of INF stock solution was used for all stress-testing investigations. The following forced degradation investigations were carried out to assess the stability-indicating properties of INF and the assessment and profiling of DPs of INF. The goal was to obtain the main DPs and minimize side reactions and further degradation of the DPs themselves.

#### 3.5.1. Acidic Hydrolysis

A 0.5 mL stock solution was used to create the sample for acid hydrolysis by adding 0.5 mL of 0.5 N HCl with a final concentration of 1.0 mg/mL. The substance was heated at 70 °C for three hours in a shaker water bath, let to drop to room temperature, and then neutralized with 0.5 N NaOH. A 0.5 mL aliquot of the aforementioned solution was diluted to a final volume of 5.0 mL using the initial mobile phase in a 5.0 mL volumetric flask. It was filtered and put into the HPLC for analysis. A blank solution was made and then processed in the exact same manner as the standard solution.

#### 3.5.2. Basic Hydrolysis

The sample for the basic degradation study was prepared in the same way as that for the acid, except that 0.1 N NaOH was used and the time in the water bath at 70 °C was reduced to one hour. The reaction was stopped with 0.1 N HCl. A blank solution was prepared and then subjected to the identical procedural steps employed for the standard solution.

#### 3.5.3. Oxidative Stress

The sample for oxidative degradation was made from a 0.5 mL stock solution by adding 0.5 mL of 2% H_2_O_2_, with a final concentration of 1.0 mg/mL. In the dark, the drug was left for six hours on a benchtop at 25 °C (room temperature). Another blank sample was prepared as a control without the drug and processed in the same way. The process was then carried out as outlined in the section on acid hydrolysis.

#### 3.5.4. Photolytic Stress

Two samples of 10.0 mg of INF were weighed in borosilicate glass. One sample was exposed to ultraviolet light (400 W·h/m^2^) and cool white fluorescent light (2.4 million Lux-hours), which is twice the ICH minimum recommended exposure. The other sample was wrapped in aluminum foil and used as a dark control. The study was carried out in a photostability chamber (APT.line^®^ KBF-ICH-720; Binder, Tuttlingen, Germany) that was outfitted with ICH-compliant option-2 illumination in the door lights and set at 40 ± 5 °C/30% relative humidity (RH) ± 3%RH. The samples were subjected to identical treatment, as previously described.

#### 3.5.5. Thermal Stress (Ambient and High Humidity)

In borosilicate glass flasks, two samples of INF weighing 10.0 mg each were weighed. One sample in an open flask over a saturated solution of sodium chloride in a desiccator was subjected to a temperature of 70 °C and 70% RH for 7 days. The other sample was treated in the same way without controlling humidity. The samples were processed in the exact same manner as was outlined in the preceding section.

### 3.6. Liquid Chromatographic Conditions

In a C_18_ column (Zorbax Eclipse Plus, 250.0 mm, 4.6 mm, 5.0 µm), HPLC separation was accomplished utilizing gradient elution and two mobile phases, A and B. Ammonium acetate buffer (mobile phase A) has a 25.0 mM concentration and a pH adjustment of 6.0. ACN is the mobile phase B. The gradient was configured as time (min)/B (%): 0.01/20, 10/40, 38/47, 48/20, and 55/20, with a flow rate of 1.0 mL/min. The column’s temperature was held constant at 25 °C. The injection volume of the standard and sample solutions was 10 µL. The absorbance was monitored at 292 nm with a photodiode array (PDA) detector.

### 3.7. LC-MS Parameters

Utilizing the Waters UPLC-MS/MS apparatus Acquity UPLC and Acquity TQD MS in conjunction with electrospray ionization (ESI) and LC-MS/MS, it was possible to determine the molecular mass of the parent compound and recently generated DPs. The cone voltage was set to 16 V, the capillary voltage to 30 V, and the probe temperature to 250 °C for the mass detector. Data collection and processing were performed using Masslynx v.4.1 software. Similar steps used in HPLC with gradient timing were used in LC-MS, except for lowering the flow rate to 0.8 mL/min to avoid overloading the mass spectrometer and decreasing the injection volume to 5 µL.

## 4. Conclusions

A stability-indicating reversed-phase HPLC method was developed for the quantification of INF, employing a QbD methodology. Prior to the development process, there was a lack of established stability-indicating analytical procedures for INF, and no INF DPs were accessible. Consequently, the utilization of forced degradation samples became a prominent strategy in the QbD framework. A mathematical framework was developed to analyze the CQAs associated with ATP. A method for designing a robust region was proposed within the control space of the design region. The utilization of a mathematical model facilitates a more comprehensive comparison of the impact of procedure parameters on outcomes. The analytical method that was designed underwent validation at the chosen working point to assess its accuracy, repeatability, sensitivity, and linearity. The approach that was created successfully achieved the predetermined acceptance criteria for ATP established at the initiation of the QbD process. The LC method that has been developed demonstrates the capability to effectively separate five distinct DPs, namely D1, D2, D3, D4, and D5. Furthermore, the methodology is expanded to include LC-MS, thereby facilitating the analysis and determination of the structural properties of diverse compounds. Additionally, in silico toxicity of INF and its DPs was evaluated with DEREK and SARAH software. Different toxicity outcomes, such as chromosomal damage, were predicted. Furthermore, it was predicted that two degradation products would cause mutations. This prediction needs further assessment with in vitro and in vivo experiments. Finally, obtaining the degradation products is a prospective future project. The approach that has been devised can be employed in quality control laboratories for the purpose of quantitatively determining and serving as the SIAM for INF.

## Figures and Tables

**Table 1:** ANOVA for quadratic model of the first response for number of peaks with a resolution ≥ 1.5.

Source	Sum of Squares	df	Mean Square	F-Value	*p*-Value	Notes
Model	45.42	13	3.49	133.10	<0.0001	significant
A-pH	0.1139	1	0.1139	4.34	0.0526	
B-Flow Rate	0.0907	1	0.0907	3.45	0.0805	
C-Temperature	0.7607	1	0.7607	28.98	<0.0001	
D-Organic Phase	35.77	1	35.77	1362.63	<0.0001	
AB	0.7345	1	0.7345	27.98	<0.0001	
AC	0.1555	1	0.1555	5.92	0.0263	
AD	0.1859	1	0.1859	7.08	0.0165	
BC	0.1906	1	0.1906	7.26	0.0153	
BD	0.0807	1	0.0807	3.07	0.0977	
CD	1.23	1	1.23	47.02	<0.0001	
A²	0.9378	1	0.9378	35.72	<0.0001	
B²	1.03	1	1.03	39.05	<0.0001	
C²	3.09	1	3.09	117.57	<0.0001	
Residual	0.4463	17	0.0263			
Lack of Fit	0.4463	8	0.0558			
Pure Error	0.0000	9	0.0000			
Cor Total	45.87	30				

**Table 2:** ANOVA for quadratic model of the second response for the total number of peaks.

Source	Sum of Squares	df	Mean Square	F-Value	*p*-Value	Note
Model	39.68	13	3.05	164.03	<0.0001	significant
A-pH	0.0056	1	0.0056	0.3019	0.5899	
B-Flow Rate	0.0018	1	0.0018	0.0965	0.7599	
C-Temperature	1.24	1	1.24	66.82	<0.0001	
D-Organic Phase	33.28	1	33.28	1788.17	<0.0001	
AB	1.70	1	1.70	91.49	<0.0001	
AC	0.0020	1	0.0020	0.1087	0.7457	
AD	0.0019	1	0.0019	0.1018	0.7536	
BC	0.0163	1	0.0163	0.8742	0.3629	
BD	0.0053	1	0.0053	0.2850	0.6003	
CD	0.7439	1	0.7439	39.97	<0.0001	
A²	0.4545	1	0.4545	24.42	0.0001	
B²	0.7120	1	0.7120	38.26	<0.0001	
C²	2.43	1	2.43	130.52	<0.0001	
Residual	0.3164	17	0.0186			
Lack of Fit	0.3164	8	0.0395			
Pure Error	0.0000	9	0.0000			
Cor Total	40.00	30				

**Table 3:** Percentage of each degradation product in different stress conditions.

Condition	D1	D2	D3	D4	D5
Acidic	11.1%	0.12%	--	0.08%	--
Basic	3.22%	0.07%	--	0.05%	--
Oxidative	3.62%	0.14%	0.005%	0.009%	5.49%
Thermal uncontrolled	3.05%	0.15%	--	--	--
Thermal controlled	2.24%	0.1%	--	--	--
Photo	3.73%	0.12%	0.014	--	--
Photo control	2.72%	0.09%	--	--	--

**Table 4:** The MS data of Infigratinib and its degradation products, accompanied with their respective potential degradation pathway and product ions in the electrospray ionization (ESI) positive mode.

ID	*m*/*z*	MS/MS Fragment Ions	Proposed Degradation Pathway
INF	560	339, 313	--
D1	313	269, 242, 214, 196	Hydrolysis
D2	441	424, 337, 297, 198	Hydrolysis
D3	201	172, 157, 124, 121, 97	Oxidation-photolytic
D4	343	240, 194	Hydrolysis
D5	576	560, 313, 230, 213	Oxidation

**Table 5:** Regression and validation parameters of the HPLC method for Infigratinib.

Parameter	INF
Linearity range (µg/mL)	5.0–200.0
Slope	20,886.63
Intercept	−121,901.93
Correlation coefficient	0.9999
Accuracy (mean ± SD)	99.55 ± 0.67
%RSD	0.67
Precision ^a^ (RSD)	1.27
Intermediate precision ^b^ (RSD)	1.44
LOD (µg/mL)	1.56
LOQ (µg/mL)	4.74

^(a)^ Intraday precision (average of three different concentrations of three replicates each (n = 9) within the same day); the concentrations were (15, 80, 130 µg/mL) of INF. ^(b)^ Interday precision (average of three different concentrations of three replicates each (n = 9) repeated on three successive days); the concentrations were (15, 80, 130 µg/mL) of INF.

**Table 6:** System suitability parameters for the optimized HPLC method.

Parameters	D1		D2		D3		D4		Infigratinib		D5		Value Reference ^a^
Resolution R_s_		4.2		3.0		54.7		8.9		4.1			R_s_ ≥ 2
Selectivity α		2.0		2.3		5.8		7.6		9.4			1 < K < 10
Tailing factor	2.1		1.8		1.2		1.2		1.7		1.0		T ≤ 2
Column efficiency (N)	2256.2		11,573.0		30,169.8		297,037.2		9564.6		5573.5		N > 2000
HETP ^b^	9024.7		46,292.0		120,679.1		1,188,148.7		38,258.3		22,294.0		

^a^ USP reference. ^b^ Height equivalent to theoretical plate (cm/plate).

**Table 7:** Robustness results of the optimized HPLC method for Infigratinib and its degradation products.

**Confirmation Location #1**									
pH	Flow Rate	Temperature	Organic Phase	Statistical Parameters					
5.9	1.1	27.3	ACN						
Response	Mean	Median	Observed *	SD	n	SE	95% low	Mean	95% high
# of peaks ≥1.5	6.03	6.03	6.00	0.16	3.00	0.12	5.77	6.00	6.29
# of peaks	5.91	5.91	6.00	0.14	3.00	0.10	5.69	6.00	6.13
**Confirmation Location #2**									
pH	Flow Rate	Temperature	Organic Phase	Statistical Parameters					
6.0	1.0	26.8	ACN						
Response	Mean	Median	Observed *	SD	n	SE	95% low	Mean	95% high
# of peaks ≥1.5	6.14	6.14	6.00	0.16	3.00	0.13	5.87	6.00	6.41
# of peaks	6.04	6.04	6.00	0.14	3.00	0.11	5.81	6.00	6.26
**Confirmation Location #3**									
pH	Flow Rate	Temperature	Organic Phase	Statistical Parameters					
6.2	0.9	26.8	ACN						
Response	Mean	Median	Observed *	SD	n	SE	95% low	Mean	95% high
# of peaks ≥1.5	5.88	5.88	6.00	0.16	3.00	0.13	5.60	6.00	6.16
# of peaks	5.79	5.79	6.00	0.14	3.00	0.11	5.55	6.00	6.02

* Average of three determinations.

**Table 8:** Toxicity and mutagenicity prediction of Infigratinib and its degradation products.

Drug/DP No.	DEREK			SARAH				
	Structural Alert Code	Structural Alert	Endpoints for Toxicity	Hypothesis		Structural Alert		Endpoint for Mutagenicity
Drug	624	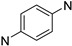	Chromosome damage	Negative				
	726	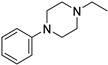	Phospholipidosis					
	918	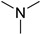	Skin irritation					
	435	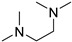	Skin sensitization					
	837	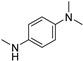						
D1	624	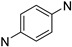	Chromosome damage	H-216	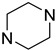		Negative	
	726	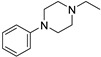	Phospholipidosis					
	918	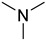	Skin irritation	H-58	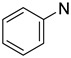		Positive	
	435	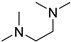	Skin sensitization					
	837	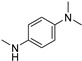		H-190	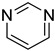		Negative	
D2	624	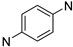	Chromosome damage	Inconclusive				
	553	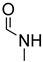	Hepatotoxicity					
	440	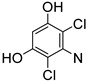	Skin sensitization					
	837	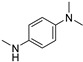						
	248	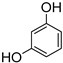	Thyroid toxicity					
D3	No alerts were found for toxicity			H-58	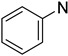		Positive	
				H-190	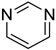		Negative	
D4	624	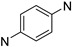	Chromosome damage	H-216	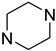		Negative	
	726	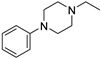	Phospholipidosis	H-58	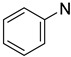		Positive	
	918	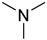	Skin irritation	H-190	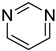		Negative	
	435	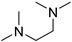	Skin sensitization	H-190	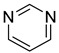		Negative	
	837	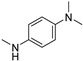						
D5	918	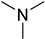	Skin irritation	Negative				

## Data Availability

All data are available within the manuscript.

## Supplementary Materials

The following supporting information can be downloaded at: https://www.mdpi.com/article/10.3390/molecules28227476/s1. Figure S1: Chromatograms that has been chosen from the results acquired through DoE following the optimization process from the sample mixture, acid base, oxidation, thermal uncontrolled, thermal controlled, photo, and photo dark control samples; Figure S2: ESI-MS fragmentation spectrum of ([M + H]^+^) ion of D1 (*m/z* 313); Figure S3: ESI-MS fragmentation spectrum of ([M + H]^+^) ion of D2 (*m/z* 441); Figure S4: ESI-MS fragmentation spectrum of ([M + H]^+^) ion of D3 (*m/z* 201); Figure S5: ESI-MS fragmentation spectrum of ([M + H]^+^) ion of D4 (*m/z* 343); Figure S6: ESI-MS fragmentation spectrum of ([M + H]^+^) ion of D5 (*m/z* 576); Figure S7: Purity plot of Infigratinib.
