# Supplementary material for: Selective Stability Indicating Liquid Chromatographic Method Based on Quality by Design Framework and In Silico Toxicity Assessment for Infigratinib and Its Degradation Products
Source: Molecules. 2023 Nov 8;28(22):7476. doi: 10.3390/molecules28227476 (PMC10673276; doi:10.3390/molecules28227476)
Supplement: Supplementary file 1 [file molecules-28-07476-s001.zip › molecules-2643385-supplementary.pdf]

<u>Supplementary Information</u>

Selective Stability Indicating Liquid Chromatographic Method Based on Quality by Design framework and in silico toxicity assessment for Infigratinib and Its Degradation Products

Awadh M. Ali, Mohammed M. Alanazi, Mohamed W. Attwa, Hany W. Darwish

Department of Pharmaceutical Chemistry, College of Pharmacy, King Saud University, P.O. Box 2457, Riyadh 11451, Saudi Arabia

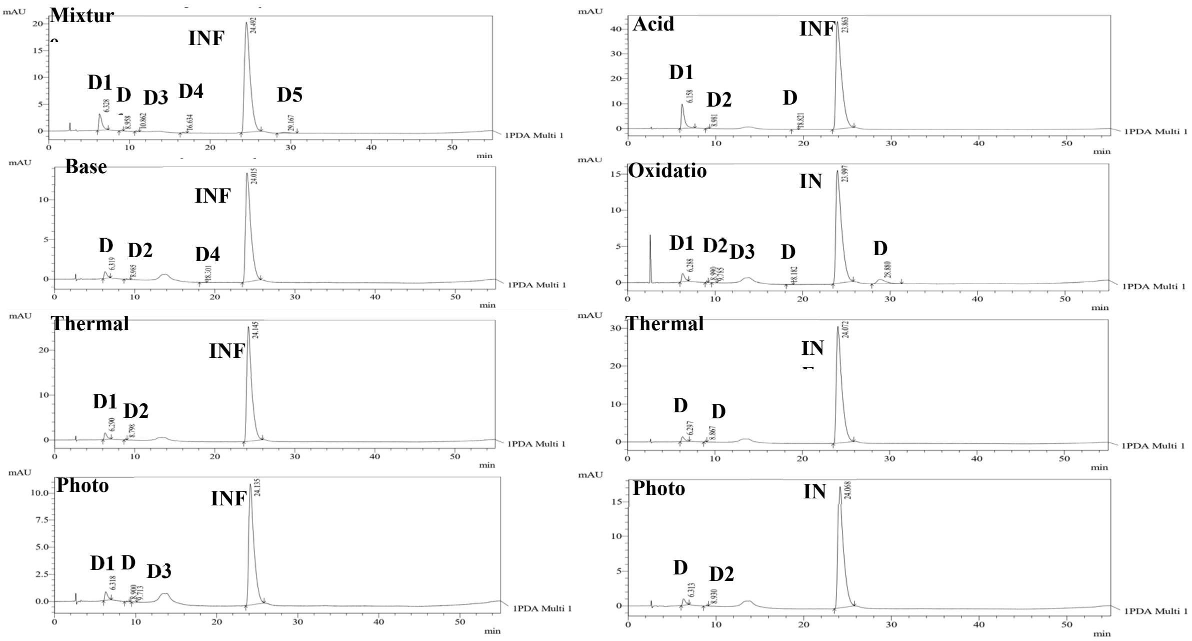

Figure S1: Chromatograms that has been chosen from the results acquired through DoE following the optimization process from the sample mixture, acid base, oxidation, thermal uncontrolled, thermal controlled, photo, and photo dark control samples.

I. MS/MS fragmentation pattern of DPs of INF

The MS spectra of the primary DP (D1) exhibit the presence of the ($[M + H]^+$) ion peak at m/z of 313, accompanied by an elemental formula of $C_{17}H_{25}N_6^+$. The rationale for the suggested structure was supported by its fragmentation pathway, as seen in Figure S1. The fragmentation spectra of the protonated molecular ion ($[M + H]^+$) of D1 exhibited distinct product ions at m/z 269, m/z 242, m/z 214, and m/z 196. These observed ions played a crucial role in the determination of the suggested structure for D1.

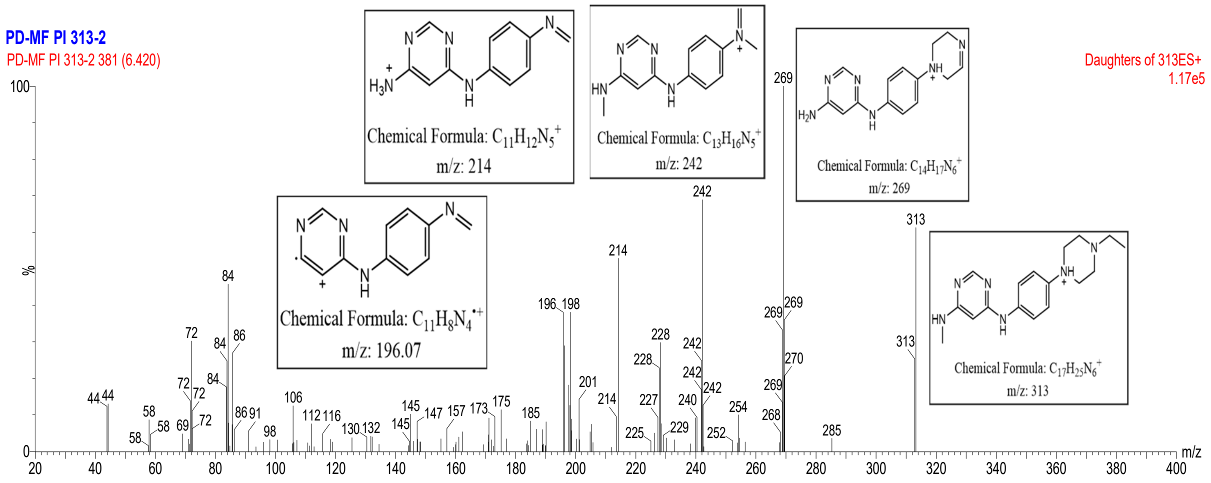

Figure S2. ESI-MS fragmentation spectrum of ($[M+H]^+$) ion of D1 (*m/z* 313).

The fragmentation spectrum of the compound $C_{17}H_{19}Cl_2N_6O_4^+$ (D2) exhibited distinct product ions with m/z values of 424, 337, 297, and 198, as seen in Figure S2. The ions had a significant role in determining the probable structure that could be postulated.

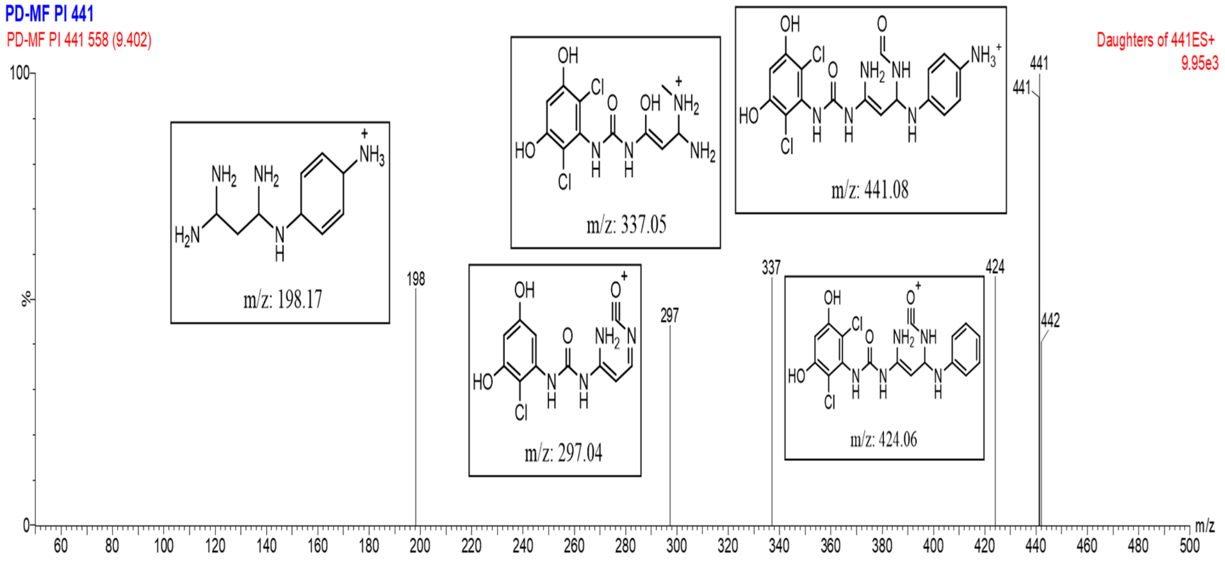

Figure S3. ESI-MS fragmentation spectrum of ($[M+H]^+$) ion of D2 (*m/z* 441).

The mass fragmentation spectrum of the ($[M + H]^+$) ion (m/z 201) of D3 and its elemental formula ($C_{11}H_{13}N_4^+$) exhibit product ions at m/z 172, 157, 124, 121, and 97 (Figure S3). Based on the findings presented, it was proposed that D3 corresponds to N^4-methyl-N^6-phenylpyrimidine-4,6-diamine.

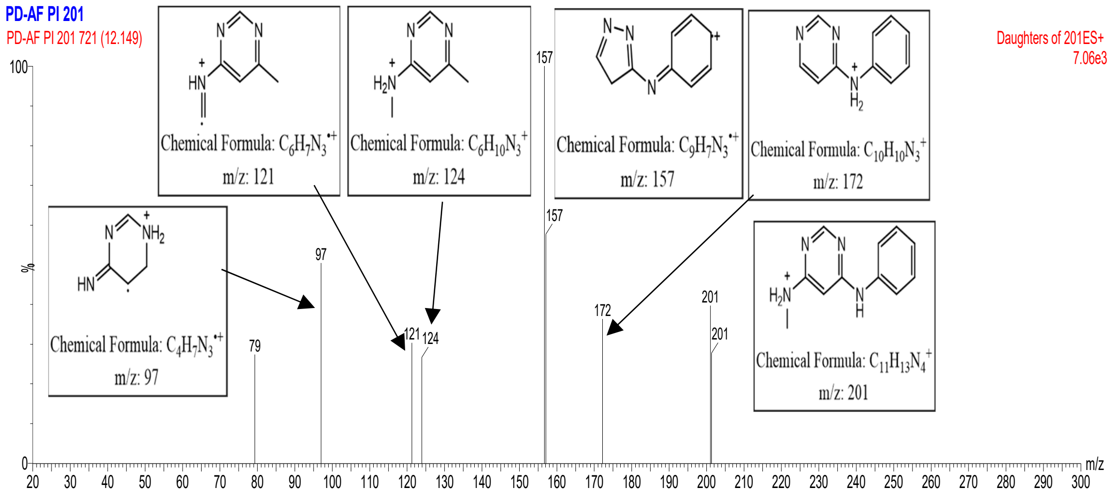

Figure S4. ESI-MS fragmentation spectrum of ($[M+H]^+$) ion of D3 (*m/z* 201).

The molecular structure proposed for D4 was derived from m/z measurements of the MS/MS product ion spectrum, and the proposed structure is indicative of product ions at m/z 240, and 194 as shown in Figure S4. The most probable structure of D4 was proposed as (6-((4-(4-ethylpiperazin-1-yl)phenyl)amino)pyrimidin-4-yl)carbamic acid.

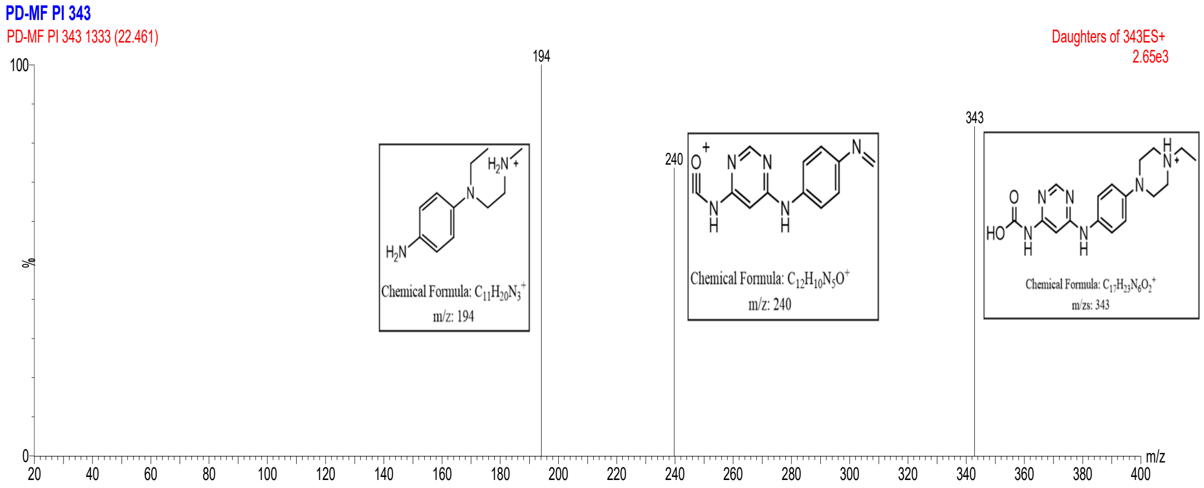

Figure S5. ESI-MS fragmentation spectrum of ($[M+H]^+$) ion of D4 (m/z 343).

The degradation product D5, which is an N-oxide, has a fragmentation pattern characterized by m/z values of 576 and 592, indicating twofold oxidation. This fragmentation process yields m/z values of 560 (corresponding to the parent molecule), 313 (which comprises a fragment of the parent compound), 230, and 213, as seen in Figure S5. The suggested structure is referred to as 1-(4-((6-(3-(2,6-dichloro-3,5-dimethoxyphenyl)-1-methylureido)pyrimidin-4-yl)amino)phenyl)-4-ethylpiperazine 1-oxide, which has a chemical formula of $C_{26}H_{31}Cl_2N_7O_4$.

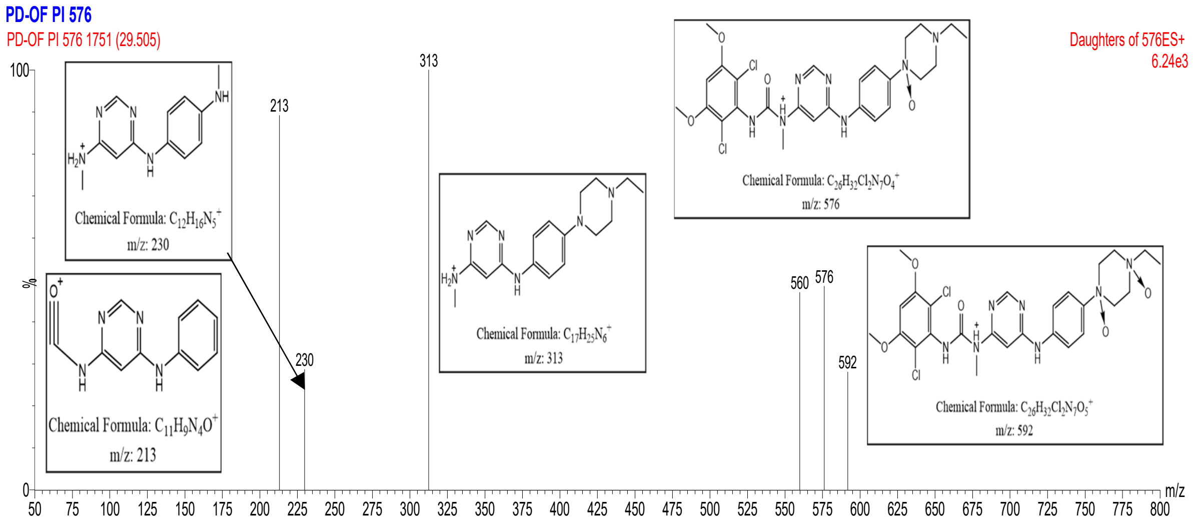

Figure S6. ESI-MS fragmentation spectrum of ($[M+H]^+$) ion of D5 (m/z 576).

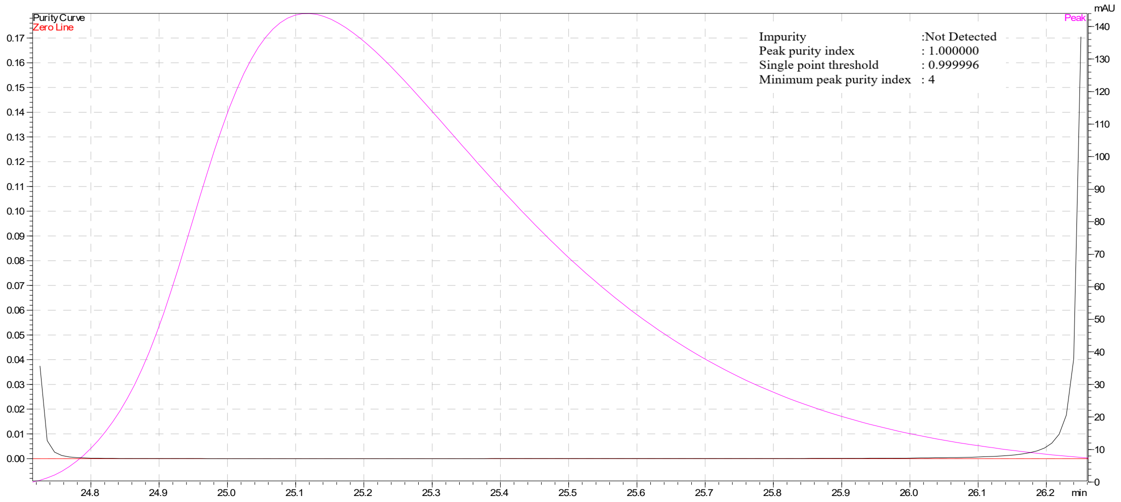

Figure S7: Purity plot of Infigratinib.
